# The Unique Features of Pediatric Sports Injuries: The Hip

**DOI:** 10.3390/children13091229

**Published:** 2026-09-11

**Authors:** Leah Henry, Karli Funk, Yi-Meng Yen, Joshua Abzug

**Affiliations:** 1 Department of Orthopaedics, University of Maryland School of Medicine, Baltimore, MD 21201, USA; karlifunk@som.umaryland.edu (K.F.);; 2 Department of Orthopaedics, Boston Children’s Hospital, Waltham, MA 02115, USA

**Keywords:** pediatrics, adolescents, orthopaedics, sports injuries, lower extremity, hip, femoroacetabular impingement syndrome, stress fractures, overuse injuries

## Abstract

**Highlights:**

**What are the main findings?**

- This narrative review summarizes the clinical presentation of apophyseal injuries, hip dislocation, coxa saltans, femoroacetabular impingement syndrome (FAIS), and stress fractures.
- Evidence-based diagnosis, management, and complications of these conditions are reviewed.

**What are the implications of the main findings?**

- While apophyseal injuries, coxa saltans, and FAIS should be managed based on symptomatic presentation, hip dislocations and femoral neck stress fractures require timely diagnosis and reduction to prevent further complications.
- The musculoskeletal care provider should be an active member of the interdisciplinary team when assessing the pediatric athlete and determining appropriate management and return to sport.

**Abstract:**

Sports-related injuries of the hip and groin have demonstrated increasing prevalence in pediatric and adolescent athletes. Pediatric injuries require specific consideration of unique factors such as skeletal maturity, apophyseal vulnerability, physeal closure status, and growth-related changes in injury pattern. These injuries include a variety of conditions, including apophyseal injuries, hip dislocations, coxa saltans (snapping hip), femoroacetabular impingement syndrome (FAIS), and stress fractures. Injuries such as hip dislocation and high-risk femoral neck stress fracture require urgent diagnosis and management; however, timely diagnosis and appropriate management is necessary for all pediatric hip injuries to prevent delays in return to sport and long-term complications. The literature synthesized in this narrative review includes randomized controlled trials, systematic reviews, prospective and retrospective studies, and consensus statements to provide a summary of each injury. An overview of pediatric sports injuries of the hip, with emphasis on the epidemiology, presentation, diagnosis, management, and complications, is presented.

## 1. Introduction

The incidence of sports-related injuries in adolescent and pediatric athletes has increased as involvement in competitive and organized sports has risen [1]. Common sports-related hip and groin injuries in pediatric and adolescent patients encompass a variety of conditions, including apophyseal injuries, hip dislocations, coxa saltans (snapping hip), femoroacetabular impingement syndrome (FAIS), and stress fractures [2,3,4]. A comprehensive understanding of the most prevalent pediatric sports injuries is essential for the musculoskeletal care provider [3,4]. While the prior literature has presented comprehensive overviews of upper- and lower-extremity pathologies in the pediatric athlete, there is limited literature dedicated to comprehensively describing pediatric injuries of the hip as they pertain to the orthopedic provider. This review aims to describe the relevant clinical presentation, management, complications, and prevention for selected sports-related pediatric hip injuries.

## 2. Methods

The purpose of this review is to present a comprehensive, educational overview of pediatric hip injuries in athletes for the musculoskeletal care provider. As defined by the American Academy of Pediatrics, pediatric patients are defined as those under the age of 21 and adolescents are those between the age of 11–21 [5]. Conditions were included based on prevalence and relevance to sport participation. Databases searched included Pubmed and Scopus. Publications eligible for inclusion were systematic reviews and meta-analyses, randomized controlled trials, prospective and retrospective studies, and consensus statements published by academic societies in the date range from 2000 to 2026 to ensure a comprehensive but contemporary overview. Editorials and non-peer-reviewed conference articles were excluded. Studies included pediatric patients and adolescents up to the age of 24, with incorporation of evidence from the literature on adults when pediatric data was not available. Peer-reviewed articles were qualitatively appraised by two authors with prioritization given to those of the highest tier evidence. OpenEvidence (an LLM-based clinical evidence tool; Cambridge, MA, 2026) was used in conjunction with this process to identify additional relevant references. References suggested by OpenEvidence were verified for accuracy, located independently within the primary literature, and screened against the predefined inclusion criteria.

## 3. Applied Anatomy of the Developing Hip

The pediatric hip differentiates from the lower-limb bud mesoderm to resemble an adult hip by 8 weeks of gestation [6]. Further development of the hip is then driven postnatally by growth of both the acetabulum and the proximal femur from their respective epiphyseal plates [6,7]. The hip joint consists of the concave acetabulum, which articulates with the spherical femoral head in a diarthrodial joint [8]. The acetabulum comprises the ilium, ischium, and pubis, with a lateral cup shape and medial tri-radiate formation [9]. Although individual variability exists, ossification centers usually appear at age 10–11 in females and 13–14 in males and close sequentially at approximately age 13–14.5 in females and 14 in males [10]. Femoral growth is driven by appositional growth at the femoral head and greater trochanter and from the epiphyseal plate at the femoral neck [11]. Variations in male and female hip anatomy are evident in the acetabular index and medial joint space [12]. Pediatric athletes are predisposed to musculoskeletal injuries of the physes, as these function as areas of decreased mechanical strength. Apophysitis and epiphyseal injuries can occur at these regions in the setting of repetitive mechanical stress during sport [13].

Abnormal developments in bony morphology of the hip usually occur during early adolescence. Cam morphology, which is the abnormal development of the femoral head–neck junction, starts to develop between the ages of 10–14, prior to the closure of the proximal femoral physis around ages 16–18 [14]. This abnormal development is primarily driven by the capital femoral growth plate extending in the anterosuperior direction into the femoral neck during development, thereby decreasing the femoral head–neck offset and increasing the alpha angle [15]. In contrast, pincer morphology is defined as overhanging of the acetabulum and may arise during adolescence as acetabular ossification progresses and abnormal version changes occur around the time of triradiate cartilage closure [16]. Abnormal morphology alone may be asymptomatic, or can result in pathologic femoroacetabular impingement (FAI) [17]. FAI syndrome is defined by symptoms of clicking, stiffness, or clicking, clinical signs such as pain or restricted range of motion with examination, and radiographic evidence of cam or pincer morphology [18].

The hip joint relies on static stability from the labrum, capsule, capsular ligaments, and ligamentum teres, as well as dynamic stability from hip musculature [19]. The joint capsule consists of longitudinal fibers that form capsular ligaments—the iliofemoral, pubofemoral, and ischiofemoral ligaments—as well as circular fibers that form the zona orbicularis that encircle the femoral neck [19,20]. The ligamentum teres houses the foveal artery, which provides femoral head perfusion during infancy and early childhood. In addition to its vascular function, the ligamentum teres acts as a secondary constraint during high flexion, adduction, and external rotation of the hip [21,22].

The arterial supply of the hip develops in accordance with further musculoskeletal growth [21]. The pediatric femoral head is predisposed to avascular necrosis when physes are open, as this creates a functional barrier between the epiphyseal and metaphyseal circulation. While the physis remains open, the femoral head is supplied largely by the lateral epiphyseal vasculature [23]. At age 3 months and below, the femoral head is supplied by the ligamentum teres and lateral epiphyseal arteries. By 18 months, the lateral epiphyseal arteries, which are branches of the medial femoral circumflex artery, become the dominant source of arterial supply [23]. By adolescence, there are metaphyseal and epiphyseal circulations that supply the femur. The medial femoral circumflex artery becomes the dominant supply to the femoral head in adulthood [24].

## 4. Physical, Psychosocial, and Financial Impact of Pediatric and Adolescent Sports-Related Hip Injuries

Sport-specific hip injuries require comprehensive examination and evaluation by a qualified musculoskeletal care provider to ensure prompt diagnosis and reduce the risk of subsequent complications [3,4]. Time away from sport varies significantly based on injury and operative vs. non-operative treatment pathways [25]. Returning to play following sport-related injury requires comprehensive interdisciplinary care between physician, athlete, physical therapist, and coach [4,26]. The Team Physician Consensus relies on a process of physician-led shared decision making to optimize return-to-sport trajectory and timeline [27]. Delayed or improperly diagnosed pediatric or adolescent hip conditions, such as spontaneously reduced hip dislocations, femoral neck stress fractures, and apophyseal avulsion fractures, can result in long-term functional complications [28,29,30,31,32].

Sports injuries of the adolescent athlete are multi-factorial and involve complex socioeconomic factors; thus, the psychosocial impact of injuries limiting sport participation cannot be ignored. Youth athletes may face challenges such as social isolation, depression, and anxiety following athletic injuries, thereby warranting the involvement of mental health professionals to optimize return to sport [33]. Kinesiophobia or fear of reinjury may limit return to sport following injury, particularly in those who identify strongly as athletes [34,35]. Early interventions such as cognitive behavioral therapy and stress management may reduce the burden of such factors, but these methods remain understudied in the pediatric population [35,36].

While data specific to hip injuries in pediatric patients is limited, pediatric and adolescent sports injuries collectively have critical economic implications and result in nearly 2.7 million emergency department visits annually [37]. In a 4-year period, the estimated total hospital charges for pediatric sports injuries were $485 million [38]. Although there are currently no studies specifically investigating the financial burden of pediatric sports-related hip injuries, these types of financial studies have been conducted in athletes with other types of injuries. Significant treatment-related financial burden has been reported by families of pediatric athletes undergoing anterior cruciate ligament (ACL) reconstruction, for example, with 58% of families experiencing economic burden related to the injury [39]. Though these findings cannot be linearly extrapolated to pediatric hip injuries, they underscore the significant financial burden of sports-related injuries across the pediatric and adolescent populations.

## 5. Epidemiology of Pediatric and Adolescent Sports-Related Hip Injuries

Injuries of the hip and groin represent 11% of all sports injuries in athletes age 12–28, with a seasonal prevalence of 19% across all age ranges according to a recent meta-analysis of 5914 hip and groin injuries [40]. While pediatric-specific epidemiological data is lacking, a period of increased risk for overuse injury in adolescent athletes age 10–19 is present at the time of peak height velocity, which typically occurs at age 11.5 in girls and 13.5 years in boys [41]. This time frame has been correlated with heightened risk of hamstring and groin injuries in particular [41,42]. For example, in youth elite soccer players, hip and groin injuries have the greatest total injury burden (incidence × severity) among all lower-limb injury locations [25,43].

The incidence of hip and groin injuries in pediatric athletes has been associated with male sex [40,44]. Pediatric and adolescent male athletes have a 1.8–2.3 times greater risk of hip injury [40,44]. In particular, male athletes have a higher incidence of avulsion fractures [45]. However, female athletes have demonstrated a greater propensity for soft tissue and overuse injuries, such as labral tears, tendonitis, and coxa saltans [45]. Differences in bony morphology, muscular recruitment, and sport selection may contribute to such findings [46,47,48]. These sex-based differences underscore the importance of tailored screening and prevention strategies for male and female adolescent athletes.

## 6. Differential Diagnosis of the Painful Pediatric or Adolescent Hip

When evaluating a painful hip in a pediatric or adolescent athlete, other important causes of a painful hip unrelated to the sport must be ruled out in the meantime. The non-athletic diagnoses to consider include a wide variety of conditions, including transient synovitis, septic hip, osteomyelitis, Lyme disease, Legg–Calve–Perthes (LCP) disease, slipped capital femoral epiphysis (SCFE), juvenile idiopathic arthritis, osteonecrosis, malignancy, and referred pain from lumbar or abdominal pathologies. Ruling out these diagnoses is crucial, as many of them can have significant long-term negative effects on the health of the developing hip or the patient’s overall physical well-being if left untreated. In addition, musculoskeletal injuries of the hip and groin may present primarily with referred knee pain, making a comprehensive hip examination a critical component of any evaluation for adolescent knee pain [3]. Emergent management is warranted with evidence of red-flag symptoms, including an inability to bear weight, fever accompanied by refusal to bear weight, night pain, and neurovascular complaints, in addition to high-energy mechanisms and traumatic hip dislocation [49,50,51,52]. For the purpose of this review, we will focus more specifically on sports-related hip injuries and overuse conditions.

## 7. Common Sports-Related Hip Conditions in Pediatric and Adolescent Athletes

### 7.1. Apophyseal Injuries

#### 7.1.1. Background

Apophyseal injuries around the hip joint represent a spectrum of conditions exclusive to skeletally immature athletes, ranging from chronic apophysitis to acute avulsion fractures [53]. These injuries result from traction forces at the secondary ossification centers of the pelvis and proximal femur [4]. These secondary ossification centers follow a predictable pattern of development, with the proximal femoral centers (lesser trochanter, femoral head, greater trochanter) and anterior inferior iliac spine (AIIS) generally fusing by the mid-to-late teenage years, while the anterior superior iliac spine (ASIS), ischial tuberosity, iliac crest, and symphysis pubis are among the last to fuse and may remain open into the mid-twenties [3,53]. This prolonged window of vulnerability, combined with increasing muscle strength during puberty, places adolescent athletes at particular risk for these injuries. The average age at the time of injury is around 14 years, with the highest risk occurring in athletes aged 14–17 [3,32,54].

Apophyseal injuries commonly result from a sudden, forceful eccentric contraction during movements such as sprinting, kicking, or jumping, with the AIIS, ASIS, and ischial tuberosity representing the most commonly injured sites in adolescent patients [4,32,54]. Less commonly injured structures include the iliac crest, lesser trochanter, and pubic symphysis [55]. The highest prevalence of injuries is among soccer players, followed by football, baseball, and track and field athletes, with gymnastics as a commonly implicated sport as well [4,54,56,57]. Prior studies retrospectively examining apophyseal injuries have identified increased prevalence in male patients, representing 76–79% of cases [29,32,55].

#### 7.1.2. Clinical Presentation

Avulsion injuries typically present with the sudden onset of sharp, shooting pain localized to the affected apophysis, accompanied by weakness and an inability to continue sport participation [3,4]. Athletes frequently report feeling or hearing a “pop” at the time of injury and present with swelling or a limp on initial evaluation [3,4,55]. Physical examination reveals localized tenderness, decreased strength of the associated muscle group, and possible edema or ecchymosis at the injury site [56]. Both passive stretching and active contraction of the involved musculotendinous unit typically reproduce pain.

Apophyseal injuries can often be misdiagnosed as adductor muscular strains, as these injuries share similar mechanisms and clinical presentations. However, apophyseal injuries are much more common in the pediatric population, as the apophysis is the weakest link in the musculotendinous chain in the pediatric population [57]. Misdiagnosing apophyseal injuries as adductor strains and treating with an early or aggressive range of motion may result in adverse outcomes, underscoring the importance of an accurate and timely diagnosis in pediatric athletes [53]. Clinical correlation with patient age, mechanism of injury, and sport activity is essential to maintain a high index of suspicion for apophyseal injury in this population [53,55].

In addition, apophysitis, or chronic inflammation of the apophyseal site, must be distinguished from an acute avulsion injury. Apophysitis presents with an insidious onset of pain typically associated with a recent increase in training load, whereas avulsion injuries present acutely with a specific injury [3]. Notably, preexisting apophysitis may serve as a prodrome to acute avulsion, as chronic traction weakens the apophysis and predisposes it to acute failure [4]. Fracture site distribution has been shown to vary with skeletal maturity. Schuett et al. assessed fracture patterns relative to Risser stage and triradiate cartilage status, demonstrating that fracture location is significantly associated with the degree of skeletal maturation [32].

#### 7.1.3. Imaging

Plain radiographs are the initial diagnostic imaging modality of choice for suspected apophyseal avulsion fractures and are usually definitive in demonstrating the fracture and its displacement [3,29]. Standard views include an anteroposterior (AP) pelvis as well as an AP and lateral of the affected hip [3,4,29]. Oblique views of the pelvis may be needed if a suspected avulsion is not seen on standard views [3,4]. Views that require significant hip movement, such as a frog-leg lateral, should be considered with caution, as this motion can potentially further displace AIIS and ASIS avulsion fractures. Subacute and chronic avulsion injuries can develop an aggressive radiographic appearance with mixed lysis, sclerosis, and exuberant callus formation that may closely mimic neoplasm or infection, potentially prompting unnecessary biopsy; therefore, familiarity with healing patterns in these injuries is essential to avoid misdiagnosis [3,58]. Advanced imaging is infrequently required in these patients but may be warranted in those who present with prolonged symptoms and limited findings on plain film radiographs with high clinical suspicion [29].

#### 7.1.4. Non-Operative Treatment

The majority of apophyseal avulsion fractures, approximately 87% in the largest available meta-analysis, are successfully managed non-operatively [59]. Conservative management is recommended for avulsion fractures without displacement or with minimal displacement [3,4,53]. Initial treatment includes pain control with non-steroidal anti-inflammatory drugs (NSAIDs), rest, and protected weight bearing with crutches until acute symptoms resolve [3,4,60]. Following resolution of initial symptoms, a comprehensive rehabilitation program should be initiated, progressing through passive stretching, active range of motion, progressive resistance training, neuromuscular control, and ultimately sport-specific exercises. Full return to sport may be permitted following this progression [53,60]. Return to sport is achieved in the vast majority of conservatively treated cases, with Moeller et al. reporting a mean duration from injury to clearance for return to play of 9–10 weeks [61]. Return to play should be considered only when clinical benchmarks have been met, including pain-free range of motion, neuromuscular control, and ability to participate in sport-specific activities [62]. However, diagnostic delay significantly prolonged the return-to-play timeline in this study, underscoring the importance of prompt diagnosis.

#### 7.1.5. Operative Treatment

Operative intervention may be considered for displaced apophyseal avulsion fractures, although no validated displacement threshold has been established. Historically, the displacement cutoff of around 15–20 mm has been used to guide surgical decision making [29,32,63]. Individualized patient-specific factors must be considered when assessing the need for operative intervention. The most recent and largest systematic review by Molina et al. demonstrates that current available evidence does not establish a validated displacement threshold at which outcomes reliably differ between operative and non-operative management in adolescent patients [59]. The authors concluded that higher quality evidence is needed before displacement alone can be considered a reliable surgical indication. Therefore, treatment decisions should consider many patient-specific factors, including displacement, fracture location, patient age, functional demands, and sport-specific goals rather than relying on a single numeric cutoff.

Surgical approach and fixation strategies vary by fracture location. Ischial tuberosity fractures are typically addressed through a subgluteal approach with screw fixation, plate fixation, or suture anchor techniques [64,65]. ASIS fractures can be treated with screw fixation or suture anchors. Patients with ASIS fractures treated operatively have been shown to achieve faster return to sport, although long-term functional outcomes are comparable to conservative management [66]. AIIS fractures are most commonly managed non-operatively. However, displaced fractures or those that develop a hypertrophic nonunion can result in symptomatic subspine impingement, which can be successfully managed with arthroscopic subspine decompression [67].

#### 7.1.6. Complications

Delayed diagnosis or misdiagnosis of an apophyseal avulsion fracture can lead to further displacement, nonunion, and prolonged functional limitation [29]. The risk of nonunion is strongly influenced by initial displacement, with a 26-fold increased risk of nonunion with >20 mm of displacement [32]. Ischial tuberosity fractures carry the highest risk of nonunion and persistent pain (27.3% in surgically treated patients, and 10.9% in non-operatively treated patients) and require the longest recovery, approximately 6 months to return to sport [32,59,65]. ASIS avulsion fractures may be complicated by meralgia paresthetica due to compression of the lateral femoral cutaneous nerve by the displaced fragment or surrounding hematoma, which can occur acutely or as a consequence of delayed diagnosis [53]. If widely displaced, a fibrous union may occur, resulting in prolonged weakness, pain, and limitation in return to sport [68]. AIIS fractures managed non-operatively carry the highest rate of persistent pain with non-operative management among the anterior avulsion sites (13.8%), primarily driven by the risk of subspine impingement from callus formation [32].

#### 7.1.7. Prevention

Limited data exist to guide prevention of apophyseal avulsion fractures, and the efficacy of current prevention strategies remains unknown. Neuromuscular training (NMT), flexibility exercises, sport-specific techniques, dynamic warm-up routines, and training load monitoring should be considered for the prevention of lower-extremity injuries. However, evidence supporting the effectiveness of these measures in preventing apophyseal injuries is lacking [3]. Early recognition and treatment of apophysitis as a prodrome may reduce the risk of progression to complete avulsion fractures, highlighting the importance of educating athletes, parents, and coaches on early warning signs such as activity-related apophyseal pain [4].

### 7.2. Hip Dislocations

#### 7.2.1. Background

Pediatric traumatic hip dislocations are rare but serious injuries associated with high-energy trauma, though they can also occur with lower-energy mechanisms, particularly in younger children [69,70]. Traumatic hip dislocations in pediatric patients more commonly occur around two peaks of age: 4–8 years and 11–15 years [71]. In children under 8 years of age, dislocations occur more frequently in females and are typically caused by mild-energy trauma [71]. In older children and adolescents, dislocations are more frequently observed in males and are associated with moderate- to high-energy trauma [72,73,74]. Anatomic features such as ligamentous laxity and elasticity of the capsule and labrum permit dislocation with less force in younger patients [75,76]. Variations in bony morphology, including acetabular retroversion and decreased posterior acetabular coverage, have been associated with sports-related posterior hip dislocation in adolescents [74]. Posterior hip dislocation has also been associated with increased alpha angle and greater prevalence of cam-type FAIS morphology [77].

#### 7.2.2. Clinical Presentation

The vast majority of pediatric hip dislocations occur in the posterior direction, accounting for approximately 86–96% of cases [69]. Patients with a posterior dislocation classically present with the affected extremity held in a position of flexion, adduction, and internal rotation [78]. In the less common anterior hip dislocation, the limb is held in extension, abduction, and external rotation [78]. On examination, apparent limb shortening, restricted range of motion, and a palpable femoral head may be appreciated. Patients are typically unable to bear weight and present with significant pain [76]. A thorough neurovascular examination is essential, with particular attention to the sciatic nerve, as nerve injury complicates approximately 5% of pediatric traumatic hip dislocations and up to 10% in adults [79]. The peroneal division of the sciatic nerve is most commonly affected [79,80].

#### 7.2.3. Imaging

First-line imaging in suspected dislocation comprises an AP radiograph of the pelvis prior to reduction [81,82]. While both AP and cross-table lateral views of the affected hip are recommended, frog-leg lateral views should be avoided due to the risk of fracture displacement in the setting of an occult or known concomitant fracture [81]. On an AP view of the pelvis, a posterior dislocation will display as the femoral head being displaced superiorly and laterally relative to the acetabulum, with the femur internally rotated and adducted [78]. The lesser trochanter is obscured or diminished in profile due to the internal rotation of the femur [83]. An anterior dislocation, by contrast, shows the femoral head displaced inferiorly and medially, with the femur externally rotated, making the lesser trochanter prominently visible [78].

Immediately after reduction, AP and cross-table lateral views of the hip should be obtained to confirm successful concentric reduction [81,84]. Asymmetric widening of the joint space on AP radiographs post-reduction may indicate interposed osteochondral fragments or labral tissue [85]. In such cases, advanced imaging should be obtained post-reduction [86]. While CT is commonly used in the adult population for detecting osseous fragments and characterizing acetabular fractures, MRI is the preferred post-reduction modality in skeletally immature patients [87]. MRI also has demonstrated enhanced diagnostic capabilities in comparison to plain radiographs alone, as radiographs can miss up to 12% of spontaneously reduced dislocations in children under 8 years [85].

#### 7.2.4. Non-Operative Treatment

Non-operative management is appropriate for most pediatric traumatic hip dislocations [69,76]. The primary indication for non-operative treatment is a concentric reduction confirmed on post-reduction radiographs, in the absence of intra-articular loose bodies, interposed soft tissue, or significant associated fractures on the post-reduction MRI [69,76,77,78,85]. Closed reduction should be performed as soon as possible, with an upper limit of 6 h from time of injury to minimize the risk of AVN [88,89]. The optimal setting for closed reduction remains debated. Factors such as injury profile, concomitant fracture, and dislocation type should be considered when selecting reduction location. Simple dislocations without physeal injury, intra-articular fragments, or suspected fracture may be successfully reduced in the ED [76]. ED reduction has been shown to be safe, particularly in younger children, with one review reporting 39.6% of acute pediatric dislocations successfully reduced in the ED without complications [90]. Adequate sedation to achieve muscular relaxation is essential for successful reduction. In the ED, intravenous ketamine is the most widely used agent for pediatric procedural sedation, with propofol as an effective alternative [91,92]. Particular caution should be taken with performing reduction in the ED in the setting of known or suspected associated fracture given the risk of further displacement [78]. If closed reduction in the ED fails, reduction in the operating room under general anesthesia should be performed urgently, maintaining the 6 h target [78]. Reduction in the operating room should be prioritized for children nearing skeletal maturity or those with high-energy mechanisms and concomitant injuries or associated fractures [71,76]. Fluoroscopic guidance in the operating room provides the advantage of real-time confirmation of concentric reduction and identification of associated injuries [93].

Reduction may be performed by a qualified and trained musculoskeletal care provider under appropriate sedation or anesthesia [94]. No single technique has demonstrated superiority, and individual success rates range from 60 to 90% [95]. In younger children, most dislocations can be reduced with gentle manipulation alone [76]. In adolescents, however, particular caution should be taken, as an unrecognized physeal injury at the time of dislocation may lead to proximal femoral epiphysiolysis during the reduction maneuver [76]. If physeal injury is suspected or physeal instability is noted under fluoroscopy, open reduction in the operating room under radiographic guidance is recommended to prevent displacement [93].

Consensus on the optimal post-reduction weight bearing protocol has not been established. Expert opinion recommends a period of protected weight bearing for children undergoing successful closed-reduction; however, this varies based on age and skeletal maturity [78]. Individual patient and injury factors must be considered. Younger children are most often placed into a hip spica cast or hip abduction brace [78]. Adequate immobilization and protection from weight bearing are particularly important in children under 10 years, as re-dislocation has been reported in this age group [90,96]. In children who are older, weight bearing should be limited to toe-touch weight bearing with crutches for a period of 2–6 weeks [77].

#### 7.2.5. Operative Treatment

There are several key indications for operative management of pediatric traumatic hip dislocations [69]. Indications include failed closed reduction, recurrent dislocation after attempted non-operative management, non-concentric reduction with joint space asymmetry (suggesting entrapped labrum or osteochondral fragment), intra-articular loose bodies, associated acetabular or femoral head fractures, and neglected dislocations persisting beyond 4 weeks [78,90,97,98]. Intra-articular pathology is particularly prevalent in young athletes with hip dislocations from sports-related injuries [77]. The acetabular “fleck” sign, a small bony fragment displaced 2–3 mm from the posterior acetabular wall on post-reduction imaging, represents an osteochondral avulsion of the posterior labrum and warrants operative treatment even when closed reduction appears concentric [98].

Surgical hip dislocation (SHD) with open reduction is the preferred open technique for non-concentric reduction with entrapped labrum or osteochondral fragments or for fixation of a femoral head fracture [99]. In the largest pediatric SHD series, Novais et al. reported posterior labral avulsion in all eight patients with non-concentric reductions, with cartilaginous posterior wall fractures in three and femoral head chondral injuries in five [99]. Labral repair was performed with suture anchors in seven of eight patients, with excellent functional outcomes and no cases of AVN at a mean follow-up of 28 months. Hip arthroscopy offers an alternative, less invasive approach, although there is currently limited evidence for these procedures in the pediatric patient population following dislocation. Small avulsed fragments of the posterior capsule and acetabulum that interfere with closed reduction can be either reduced without repair, reduced and repaired, or excised arthroscopically [97]. Hip arthroscopy to address intra-articular pathology after pediatric hip dislocation has been shown in a small case series to yield satisfactory outcomes [97]. When used appropriately, hip arthroscopy is considered a safe and effective minimally invasive option for addressing intra-articular pathology secondary to traumatic hip dislocation [100,101].

Similar to non-operative management, patients undergoing operative management for traumatic hip dislocations must be protected via toe-touch weight bearing for up to 6 weeks. After SHD with trochanteric flip osteotomy, active hip abduction should be restricted for 6–8 weeks to protect the osteotomy site and allow healing of the reattached trochanteric fragment and abductor mechanism [102]. Post-operative protocols following hip arthroscopy are considerably more variable and lack standardization; however, most protocols have an initial period of foot-flat weight bearing for 2–6 weeks [77]. Rehabilitation should be tailored to the specific patient characteristics, procedures, and surgical findings.

#### 7.2.6. Complications

Pediatric hip dislocations are associated with a high rate of associated injuries and long-term adverse events, with combined associated pathologies identified in 72% of patients in a recent systematic review [69]. Recurrent dislocation is uncommon, reported in approximately 3% of cases in a systematic review of athletes, and is more frequently observed in children under the age of 10 [77,90,96]. Direct complications of hip dislocations include acetabular fractures, labral and capsular injuries, and AVN [69,71]. AVN is the most feared complication and may occur in up to 16% of pediatric patients after dislocation [69,71,89]. Delayed reduction is the most significant modifiable risk factor. Mehlman et al. demonstrated that reduction delayed beyond 6 h conferred a 20-fold increased risk of AVN compared with reduction within 6 h [89]. In neglected dislocations (≥4 weeks from injury), rates of AVN rise to around 48% [90]. Reducing the risk of AVN involves timely diagnosis and management within a 6 h time frame [89,93].

Other potential complications include nerve injury, coxa magna, and post-traumatic osteoarthritis. Sciatic nerve injury can occur from direct compression by the posteriorly dislocated femoral head. Traumatic dislocations may be complicated by sciatic neuropathy in up to 5% of pediatric patients [103]. Prolonged time to reduction increases the risk of significant nerve injury, although the reduction maneuver itself may injure the sciatic nerve [103,104]. Coxa magna, or enlargement of the femoral head, is a recognized late complication of pediatric hip dislocation and has been observed in up to 57% of neglected dislocations [105]. The pathophysiology of coxa magna is attributed to post-traumatic hyperemia and synovitis of the hip joint, and it may also develop secondary to AVN-related growth disturbance [106].

Reduction in the hip may also be complicated by epiphysiolysis [93,107]. A case series of five adolescents without evidence of initial physeal injury undergoing closed reduction with conscious sedation found that all five patients had resultant epiphysiolysis requiring subsequent operative management [108]. While general anesthesia in the operating room may reduce the amount of force required to reduce the injured hip, conscious sedation may not achieve adequate muscle relaxation to prevent epiphyseal injury [108]. As the risk of AVN following epiphysiolysis is high, reduction performed in the operating room with general anesthesia may reduce the risk of epiphyseal displacement [93,107]. However, this risk cannot fully be eliminated with general anesthesia alone, and further research is necessary to characterize the risk factors associated with epiphyseal injury during reduction.

Despite these potential complications, long-term functional outcomes of pediatric patients after hip dislocation are generally favorable. At an average follow-up of 10 years, Mehlman et al. reported that 95% of patients experienced mild or no pain, 95% had mild or no limp, and 78% continued to participate in high-demand sports [89]. Post-traumatic osteoarthritis (PTOA) is a known late complication after hip dislocation, although the incidence of PTOA in pediatric patients is less than in adults [109].

#### 7.2.7. Prevention

Though primary preventive strategies for hip dislocation in pediatric patients are limited, prevention of lower-extremity injuries in young athletes centers on neuromuscular training (NMT) programs that incorporate gluteal, hamstring, and core strengthening alongside balance, proprioceptive, and agility exercises to improve dynamic stabilization of the hip and lower extremity [110,111,112]. In addition to thorough warm-up routines and pre-season conditioning for prevention of lower-extremity injuries, NMT should be performed 2–3 times per week for a minimum of ten minutes to reduce risk of injury [112,113]. A meta-analysis of 16 trials in youth athletes demonstrated a 42% overall reduction in lower-extremity injuries with NMT, with sessions as short as 10–15 min producing effects comparable to longer sessions [112]. While these strategies may assist with functional mobility of the hip, the literature currently does not describe strategies for the prevention of pediatric hip dislocation.

### 7.3. Coxa Saltans (Snapping Hip)

#### 7.3.1. Background

Coxa saltans, also known as “snapping hip”, is a condition where a tendon about the hip joint catches on a bony prominence during movement, which causes a “snapping” sensation [114]. This snapping sensation may be asymptomatic in some individuals, but can lead to discomfort and pain, particularly with sports that involve repetitive motions of the hip. Limited research characterizes the pathology of snapping hip in pediatric populations; however, snapping hip has been well characterized in the literature on adults [114,115]. The etiology may be multi-factorial and can include external, internal, or intra-articular causes. The external form is caused by either the iliotibial band or the anterior leading edge of the gluteus maximus tendon snapping along the greater trochanter. The internal form is caused by the iliopsoas tendon snapping over the iliopectineal eminence, femoral head, or anterior hip capsule. Less common intra-articular causes include intrinsic pathology, such as labral tears or the presence of a loose body [114].

Snapping hip is prevalent in athletes who perform extreme ranges of hip motion, such as ballet dancers, soccer players, and football players [116,117]. Notably, asymptomatic snapping hip affects an estimated 5–10% of the general population; however, its prevalence is markedly higher in specific athletic populations, with up to 58% of elite ballet dancers age sixteen and above reporting painful snapping hip symptoms [115,118]. This condition is often described in adolescent populations; however, there is a paucity of data regarding the incidence of snapping hip in pediatric populations.

#### 7.3.2. Clinical Presentation

Patients with snapping hip generally present with complaints of painful snapping of the hip with range of motion. Pain is often localized to the anterior hip or laterally at the greater trochanter [114]. Patients with intra-articular pathology are more likely to describe a sensation of clicking in the hip. A physical examination can reproduce the patient’s symptomatic snapping or clicking with range of motion of the affected hip [115,119]. For the external type, the snapping can often be reproduced by having the patient move the hip from a flexed to an extended position while the examiner palpates the greater trochanter, feeling the iliotibial band or gluteus maximus tendon snap over the bony prominence [114]. The internal snapping can be reproduced during an exam by having the patient move the hip from a flexed, abducted, and externally rotated position into extension and internal rotation, which causes the iliopsoas tendon to snap over the iliopectineal eminence or femoral head [114]. Anterior groin pain or clicking from intra-articular pathology can be reproduced with a flexion, adduction, and internal rotation (FADIR) test [4].

#### 7.3.3. Imaging

Snapping hip is traditionally a clinical diagnosis, based on physical examination findings and patient reports. Plain radiographs may be utilized to assess bony morphology and rule out alternative diagnoses [116,120]. The American College of Radiology practice parameters endorse dynamic ultrasound as the optimal imaging method for snapping hip, as this allows the patient to perform movements that induce symptoms while pathology is viewed in real time [114,121,122]. In those with intra-articular clicking, MRI may be used to assess for labral integrity or investigate for other types of intra-articular pathology [114]. MRI can also be useful for confirming the external and internal types of snapping, often showing edema and possible partial tearing of the affected tendon.

#### 7.3.4. Non-Operative Treatment

Conservative management should be trialed initially in patients with symptomatic snapping of the hip, consisting of NSAIDs, physical therapy, and targeted activity modification to avoid snapping [114,115,116]. Physical therapy should emphasize neuromuscular control and both active and passive stretching of hip musculature [115,116]. A thorough non-operative trial should be undertaken prior to consideration of operative intervention [123]. Currently, there are no guidelines as far as the length of a non-operative treatment course needed prior to consideration of more invasive options for this condition. Prior to operative intervention, a corticosteroid injection in the bursa of the affected tendon (iliopsoas bursa or trochanteric bursa) or intra-articularly can provide diagnostic and prognostic value [124]. Research on adult populations has characterized negative sequelae such as rapidly destructive hip disease; however, there is limited evidence in pediatric patients [125,126]. A systematic review of steroid injections for musculoskeletal pain conditions in pediatric patients demonstrated short-term pain relief across most studies, with adverse events including localized swelling, worsening pain, and sciatic nerve block [127]. Low-quality and limited evidence supports the use of corticosteroid injections in pediatric patients for non-rheumatological musculoskeletal pain, and further studies are required to determine their utility in the management of snapping hip [127].

#### 7.3.5. Operative Treatment

Operative treatment will vary based on the location of the snapping within the hip. External snapping hip can be addressed with the release of the iliotibial band with possible partial release of the gluteus maximus fascia and insertion. Release of the iliotibial band may be performed endoscopically or through a Z-plasty [120]. Internal snapping hip may be addressed with iliopsoas tendon lengthening or release through an open or arthroscopic approach [128]. Comparative studies of open versus arthroscopic iliopsoas release are not characterized in pediatric patients. Arthroscopic iliopsoas release has demonstrated superior clinical outcomes in comparison to open procedures in a systematic review of skeletally mature patients, with surgical indications including internal snapping hip, labral tear, and iliopsoas tendinopathy after total hip arthroplasty [129]. Intra-articular causes of snapping hip can be addressed through hip arthroscopy, which is safe and effective in skeletally immature patients with appropriate indications [130,131].

#### 7.3.6. Complications

Complications following operative management of snapping hip vary based on procedure type and surgical approach. For external snapping hip, iliotibial band release carries a low overall complication rate, with snapping eliminated in approximately 95% of patients and a 3% reoperation rate; however, complete pain relief is not achieved in approximately 12% of patients [132]. Excessive release of the iliotibial band should be avoided, as it can compromise lateral hip stability. For internal snapping hip, the primary concerns following iliopsoas lengthening or release include hip flexor weakness, iliopsoas atrophy, recurrent snapping, and iatrogenic hip instability [128]. For hip arthroscopy in skeletally immature patients, the overall complication rate is low at 1.8%, with no reported cases of physeal separation, osteonecrosis, or growth disturbance [131].

#### 7.3.7. Prevention

Prevention of snapping hip in pediatric and adolescent athletes should focus on addressing modifiable risk factors, although evidence specific to snapping hip prevention remains limited. Prospective data has not depicted effective preventive measures for snapping hip syndrome. Eccentric hip abductor weakness has been identified as a significant impairment in patients with symptomatic external snapping hip, suggesting that targeted gluteal strengthening, particularly eccentric hip abduction exercises, may play a preventive role [133]. Maintenance of adequate hip flexibility through regular stretching of the iliotibial band, iliopsoas, and surrounding hip musculature is recommended to reduce tendon-bone friction and minimize the risk of symptomatic snapping [116].

### 7.4. Femoroacetabular Impingement Syndrome (FAIS)

#### 7.4.1. Background

Femoroacetabular impingement syndrome (FAIS) arises from abnormal morphology of the acetabulum and/or proximal femur, resulting in premature contact during hip motion that leads to labral and cartilage damage [17]. Cam, pincer, or mixed morphology may result in FAIS; however, morphology alone does not indicate symptomatic pathology as in FAIS. In the cam subtype, the resulting impingement is due to an aspherical femoral head with decreased head–neck offset [134]. The pincer subtype arises from acetabular over-coverage, resulting in increased femoral head coverage and damage to the labrum. Over-coverage can be due to retroversion of the acetabulum or a deepened acetabulum, known as coxa profunda [135]. While the pincer subtype is more common in asymptomatic individuals, the mixed subtype has the greatest prevalence among symptomatic individuals, particularly men [17]. Symptomatic FAIS is more common in female patients; however, male patients typically present with greater morphologic abnormalities than female patients [134]. Cam morphology is more common among male patients, while pincer morphology is more common among female patients [136].

Primary FAIS can occur before or after physeal fusion [15,17]. Cam morphology develops during skeletal maturation, most rapidly between ages 12 and 14, through epiphyseal hypertrophy, particularly on the anterosuperior aspect of the femoral head–neck junction in response to repetitive loading, with a dose–response relationship between training intensity and cam development [137]. Increased prevalence of FAIS has been observed across sports requiring increased force production and range of motion at the hip, such as soccer, basketball, ballet, ice hockey, and golf [138]. Secondary FAIS occurs in the setting of pediatric hip conditions such as slipped capital femoral epiphysis (SCFE) and Legg-Calvê-Perthes (LCP) [17].

#### 7.4.2. Clinical Presentation

In accordance with the Warwick agreement, diagnosis of FAIS requires the triad of appropriate symptoms, positive clinical signs, and confirmatory imaging findings [18]. However, radiographic thresholds as depicted in the Warwick agreement have not been validated in pediatric patients and may not be directly translated to such populations. The onset of FAIS is typically insidious and develops over time, particularly in young, athletic patients [139]. The hallmark symptom is deep, chronic, aching anterior groin pain that is exacerbated by activities involving hip flexion, such as squatting, prolonged sitting, getting in or out of a car, and sport-specific cutting and pivoting maneuvers, and is generally alleviated with rest. Athletes frequently demonstrate the characteristic “C” sign, cupping the hand over the anterolateral hip to localize their pain [139]. Mechanical symptoms, such as popping, snapping, or catching, are commonly reported and can be suggestive of associated labral pathology [139]. In adult patients with a mean age of 35, the time from symptom onset to definitive diagnosis in adult is, on average, about 3 years, with patients evaluated by an average of around four healthcare providers prior to diagnosis [139]. As the differential diagnosis for hip pathology in pediatric patients is broad, the prompt diagnosis and management of symptoms is critical.

On examination, the most sensitive provocative tests are the anterior impingement test and the flexion-adduction-internal rotation (FADIR) test, both demonstrating approximately 80% sensitivity for FAIS [140]. A positive FABER (flexion–abduction–external rotation) test is also an important provocative maneuver to help detect symptomatic FAIS when the patient experiences anterior groin pain. This test is also helpful for ruling out FAIS when the test results in only posterior pain [140]. Painful and reduced internal rotation is also a hallmark finding with reduced internal rotation in neutral position, demonstrating the highest specificity (94%) for ruling in FAIS [140]. The hip joint is typically not tender to palpation, helping distinguish intra-articular from periarticular pathology [4]. Antalgic gait may be noted in up to 73% of symptomatic patients [139,141].

#### 7.4.3. Imaging

Radiographic views to evaluate for FAIS include AP pelvis, frog-leg lateral, 45-degree Dunn lateral, and false profile views [17]. The head–neck offset can be evaluated on lateral views. The 45-degree Dunn lateral view has demonstrated high sensitivity (91%) and specificity (88%) for detecting FAIS [142,143]. Cam-type morphology is generally characterized by the alpha angle. The alpha angle is formed by two lines: (1) a line along the axis of the femoral neck through the center of the femoral head, and (2) a line from the center of the femoral head to the point where the femoral head–neck junction first deviates from the circular contour (best-fit circle) of the femoral head [142,144]. Cam morphology is typically characterized by an alpha angle of greater than 55 degrees; however, radiographic cut-off values have not been validated in the pediatric population and must be viewed in conjunction with the overall clinical presentation of the patient [144]. Although plain radiographs are commonly used to measure the alpha angle, it is best viewed on radial reformatted MRI sequences, as the abnormal morphology of the proximal femur in a cam lesion is three-dimensional and not fully captured radiographically [145].

AP pelvis radiographs indicative of pincer morphology may demonstrate acetabular retroversion, which can be identified with the crossover sign, ischial spine sign, or posterior wall sign [143]. Less commonly, a lateral center edge angle greater than 40 degrees may be observed [146]. Such radiographic parameters are utilized in the literature on adults; however, they cannot be linearly translated to adolescent populations. When intra-articular pathology is suspected in a patient with FAIS, MRI is the preferred confirmatory imaging modality for assessment of loose bodies and chondrolabral lesions [145]. Recent evidence suggests that 3T conventional MRI may be equivalent to or even superior to a 1.5T MR arthrogram for detecting labral tears and cartilage defects, potentially reducing the need for an invasive intra-articular injection [147].

#### 7.4.4. Non-Operative Treatment

Symptomatic patients with FAIS should initially undergo a trial of conservative management [148]. Asymptomatic patients with FAIS morphology on imaging but no evidence of pain on history or exam do not require any treatment, as current evidence does not support prophylactic intervention [18]. Initial conservative management should include activity modification to avoid impingement-provoking positions such as deep flexion, squatting, and heavy lower-extremity weight lifting, along with low-dose, short-term NSAIDs for pain control as necessary [4]. As symptoms decline, supervised physical therapy emphasizing core stabilization, hip strengthening, reduction in anterior pelvic tilt, and hip mobilization should be introduced, as these active and core-focused programs have demonstrated superior functional outcomes compared with passive or unsupervised approaches [4,149]. In a prospective cohort of 93 hips, Pennock et al. found that 82% of adolescent patients were successfully managed non-operatively with removal from sport for six weeks, followed by rest, physical therapy, and activity modification, with significant improvements in outcome scores at a mean follow-up of 2 years. Physical therapy exercises largely consisted of core strengthening and minimization of deep hip flexion [148]. A subsequent study by Zogby et al. demonstrated that these improvements persisted at a mean 5-year follow-up, with no significant decline in patient-reported outcomes between the 2- and 5-year time points [150].

#### 7.4.5. Operative Treatment

Following failed conservative management, open or arthroscopic surgery may be used for the management of FAIS [141]. Open surgery may involve osteochondroplasty of the femur, acetabular rim, or both to correct impingement morphology, and retains a role in cases with a complex pathology such as concurrent dysplasia [151]. Arthroscopic osteochondroplasty with or without labral repair is the most commonly employed surgical technique, allowing for the correction of cam and pincer deformities with comparable efficacy to open approaches with fewer complications [151].

Although comparative data is lacking in the pediatric population, multiple RCTs in the adult population support the short-term superiority of hip arthroscopy over conservative care. The UK FASHIoN trial, conducted in adults older than 16 with a mean age of 35.4, demonstrated a clinically significant 6.8-point improvement in iHOT-33 favoring arthroscopy, and the FAIT trial, with a mean age of 36, showed a 10.0-point improvement in HOS-ADL favoring surgery [151,152,153]. A meta-analysis of three RCTs confirmed statistically superior outcomes for arthroscopy compared with physical therapy alone in the short term [154]. At longer-term follow-up, Husen et al. demonstrated lower rates of osteoarthritis progression in arthroscopically treated patients (26.5%) compared with non-operatively treated patients (35.2%) at a mean of 12.5 years, though conversion to total hip arthroplasty did not differ significantly between groups [155]. Although these findings cannot be directly applied to pediatric patients, these studies may relate to future sequelae of pathology of symptomatic FAI diagnosed in adolescence.

#### 7.4.6. Complications

Pediatric and adolescent patients with FAIS may experience early degenerative changes, including labral tears and cartilage damage [156,157]. In a prospective cohort of adolescent athletes with limited range of hip motion, Wyles et al. found that 62% had abnormal MRI findings at baseline, increasing to 95% at 5-year follow-up, with 27% progressing from Tönnis grade 0 to grade 1 [156]. The natural history of FAIS is associated with early degenerative changes including decreased hip internal rotation, positive anterior impingement sign, decreased hip flexion, increased alpha angle, and presence of a cam lesion. In adult patients, long-term data suggest that hip arthroscopy may slow osteoarthritis progression, as stated above; however, a statistically significant difference in conversion to THA has not been observed [155]. Surgical complications associated with hip arthroscopy occurred in approximately 3.3% to 8.3% of patients with a mean age of 30.5 years, with lateral femoral cutaneous nerve injury as the most common complaint [158]. Emerging long-term data for hip arthroscopy in adolescents are encouraging: Domb et al. and Danilkowicz et al. each reported 100% THA-free survivorship at minimum 10-year follow-up following hip arthroscopy, with adolescents achieving superior patient-reported outcomes compared with matched adult cohorts [159,160]. Nevertheless, longer follow-up beyond 10 years is necessary to determine lifetime conversion rates to THA in this young population, given their remaining decades of life expectancy.

#### 7.4.7. Prevention

The prevalence of cam morphology is greatest in male athletes, especially those who engage in high-level power sports that involve significant biomechanical stress on the hip [161,162]. Fernquest et al. highlighted that peak development of cam morphology in elite youth soccer players occurs between the ages of 11–12 years old [162]. In sports such as ice hockey, cam morphology develops during the ages of 13–16 years [161]. Secondary prevention may be achieved by avoiding exacerbating positions such as deep squatting, correcting pelvic tilt and lumbar spine mechanics, and screening high-risk athletes, although these measures are not fully supported in the literature to date [4].

### 7.5. Stress Fractures

#### 7.5.1. Background

Bone stress injuries (BSIs) occur due to repetitive training load and are typically prevalent in military recruits and endurance athletes [30]. BSIs occur on a spectrum, in which stress reactions occur due to repetitive loading and may be graded as low or high, and stress fractures occur with eventual bony deformity and discontinuity of the cortex [163,164]. Stress fractures occur with greater prevalence during the adolescent growth spurt, and a decrease in age-adjusted bone mineral density before peak height velocity may contribute [165]. In the hip and pelvis in particular, the femoral neck and sacrum are recognized as high-risk sites for BSIs [3]. The femoral neck is especially vulnerable to stress injury due to the repetitive loading that can occur during endurance activities [30]. With repetitive loading, an imbalance between osteoclast resorption and osteoblast formation results in the formation of weakened bones and stress-induced injury, which may lead to subsequent fracture [166]. Risk factors for femoral neck stress fractures (FNSFs) include vitamin D deficiency, secondary hyperparathyroidism, and poor bone mineral density [30]. Relative Energy Deficiency in Sports (REDs) has also been found to be associated with FNSFs and must be considered as a significant risk factor for BSIs when examining adolescent athletes [167]. Several bony abnormalities are associated with the risk of BSIs [30]. Coxa vara, or a femoral neck shaft angle less than 120 degrees, may increase the risk of FNSFs. Coxa valga, or a femoral neck shaft angle of greater than 135 degrees, may increase the risk of femoral head stress fractures [168].

#### 7.5.2. Clinical Presentation

Patients with BSIs will present with an insidious onset of activity-related pain over the affected bone that gradually progresses from occurring only during activity to also occurring at rest. Pain location varies by stress fracture site: femoral neck stress fractures typically localize to the anterior groin, buttock, or lateral hip, particularly at night and with weight bearing, while sacral stress injuries localize to the sacral and gluteal region [3]. Nonspecific findings, such as antalgic gait and limited ability to bear weight, may be present on examination [30]. Often, there is an accompanying history of recently increased training volume or intensity [30].

During a physical exam, point tenderness directly over the affected bone is the most consistent finding for BSIs in readily accessible areas such as the tibial diaphysis; however, BSIs of the femoral neck may present as more diffuse pain that cannot be localized with point tenderness alone [164]. For the femoral neck, other hip-specific exam maneuvers such as the FADIR and FABER tests, log roll, hop test, heel percussion, and fulcrum test will likely be more useful [3]. Importantly, the physical examination for BSIs can be nonspecific, and a high index of suspicion should be maintained in any young athlete presenting with hip or groin pain, particularly in the setting of increased training load or risk factors for REDs [30].

#### 7.5.3. Imaging

Plain radiographs of the pelvis should first be obtained, including AP pelvis, AP hip, and cross-table lateral [30]. Fractures of the superolateral femoral neck are referred to as tension-type fractures, as they lack stability and may result in displacement [166]. In contrast, medial-sided compression-type fractures may be managed non-operatively with a period of non-weight bearing followed by gradual return to weight bearing [166]. If X-rays do not demonstrate a stress fracture and the index of suspicion remains high, an MRI should be obtained [165,166]. In a systematic review of imaging modalities for lower-extremity stress fractures, in comparison to plain radiographs, which range in sensitivity from 12% to 56%, MRI had a sensitivity from 68 to 99% [169]. MRI demonstrates linear T1 and T2 hypointense signals representing fracture lines, and T1 hypointense and T2 hyperintense signals in surrounding bone marrow representing edema [28].

#### 7.5.4. Non-Operative Treatment

While many pediatric patients can be managed non-operatively or with rest, the management of FNSFs should be based on imaging findings and patient factors. Bernstein et al. proposed an MRI-based algorithm in which compression-sided fractures involving <50% of the femoral neck width on MRI examination were managed conservatively with 6 weeks of non-weight bearing [30]. While this algorithm was proposed for adult patients, the American Medical Society for Sports Medicine supports the use of imaging-guided management in their position statement on overuse injuries [165]. Patients managed conservatively who demonstrate continued symptoms after the 6-week period should undergo a repeat MRI. Fracture progression on interval imaging may warrant prophylactic surgical fixation [30,170].

#### 7.5.5. Operative Treatment

Tension-sided femoral neck stress fractures are classified as high risk regardless of fracture line visibility, as they are inherently unstable and prone to displacement [165]. These injuries, therefore, warrant urgent orthopedic referral and may require prophylactic surgical fixation even for lower-grade injuries. Pediatric guidelines lack concrete comparative evidence to support a specific surgical construct for FNSFs; however, the most commonly used implants for pediatric femoral neck fractures include three cannulated screws in an inverted triangle position or cannulated screws with pediatric femoral locking plates [171]. The operative construct selected should be based on individualized factors such as size, skeletal maturity, and fracture pattern, as well as surgeon preference. Complete, displaced fractures may require closed or open reduction. Post-operative protocols lack standardization; however, available evidence suggests weight bearing walking may begin as early as 6 weeks following surgical fixation, with gradual progression over a period of up to 3 months [172]. However, there is a paucity of high-quality literature on rates of return to activity following FNSF fixation, underscoring the need for individualized post-operative rehabilitation.

#### 7.5.6. Complications

When compared to other BSI locations, FNSFs have a relatively high-risk of complications and thus should be carefully followed clinically [173]. FNSFs are a high-risk injury warranting prolonged recovery time and individualized return-to-sport protocols to prevent further musculoskeletal injury [173]. Tension-sided fractures are at risk of displacement, nonunion, and avascular necrosis in adolescent patients [165]. Displaced fractures demonstrated significantly increased rates of developing AVN (4.8%) and nonunion (9.1%) compared to nondisplaced fractures in a population of young adults with a mean age of 21 [30,174]. Thus, prompt diagnosis and classification of fracture type is necessary to reduce the risk of further harm.

#### 7.5.7. Prevention

Stress fractures are associated with relative energy deficiencies [175]. Decreased energy intake results in decreased bone quality and health, resulting in increased vulnerability to fracture [3]. In female adolescents, vitamin D intake has demonstrated an inverse relationship with stress fractures [176]. Furthermore, female athletes are uniquely impacted by the female athlete triad, including issues with energy availability, menstrual function, and bone mineral density, which may serve as a risk factor for BSI development [2]. While REDs more commonly occurs in female athletes, athletes of all genders can be affected by this condition [177]. Established risk factors for stress fractures in adolescent athletes include BMI < 19 and prior participation in gymnastics or dance for females, and prior fracture and increasing number of running seasons for males [178]. Modifiable risk factors are sport-specific and include repetitive loading, as in running, and rapid increases in training volume [178,179]. Further studies on REDs education and stress fracture prevention are needed in the pediatric population. However, educating patients and their family members on proper sport-appropriate nutrition, caloric/energy intake, and avoidance of overtraining may prevent REDs from developing [179]. Additionally, acutely elevated training loads have shown an association with overuse injury. Appropriate training load progression, adequate recovery, and adherence to developmentally appropriate training loads may reduce the risk of this injury, though further work is needed to identify specific prevention strategies [179].

## 8. Key Takeaway Points

- Open physes around the hip can predispose young athletes to specific acute and overuse injuries. Prompt recognition, diagnosis, and management can reduce long-term complications and time away from sport.
- Common sports-related hip injuries in this population include apophyseal injuries, hip dislocations, coxa saltans (snapping hip), femoroacetabular impingement syndrome (FAIS), and stress fractures. Non-operative management of both apophyseal injuries and FAI is generally effective; however, prolonged or worsening symptomatology may warrant surgical intervention. Dynamic ultrasound should be used to examine symptomatic snapping hip. Hip dislocation is an orthopedic emergency that should be emergently addressed to prevent avascular necrosis. Tension-sided femoral neck stress fractures should be carefully assessed and operatively managed to reduce the risk of displacement and avascular necrosis.
- Interdisciplinary management of a youth athlete with physician, coach, and parental oversight is an integral component of injury prevention. Preventive measures such as titration of training load in an age-accordant fashion, adequate nutrition, and appropriate interdisciplinary oversight may reduce the risk of injury.

## 9. Summary

This article presents a narrative review of sport-specific pediatric injuries including apophyseal injuries, hip dislocations, snapping hip, FAIS, and stress fractures. These conditions arise frequently in young athletes due to the unique vulnerability of the developing musculoskeletal system, where open physes, evolving bony architecture, and repetitive sport-specific loading create a predisposition to both acute and overuse injuries. This review summarizes the current literature; however, significant gaps exist in comparative studies evaluating optimal operative and non-operative interventions for these conditions in pediatric athletes. Where research was not readily available, studies evaluating such conditions in adults has been included in a limited capacity. Early recognition of these injuries is critical, as delayed or missed diagnoses can lead to long-term complications such as chronic pain, labral damage, and premature degenerative joint disease. Musculoskeletal care providers should therefore maintain a high index of suspicion and pursue timely diagnostic evaluation and management to optimize outcomes, minimize time away from sport, and reduce the risk of long-term sequelae.

## Data Availability

No new data were created or analyzed in this study. Data sharing is not applicable to this article.
